# Identifying geographical inequalities of maternal care utilization in Ethiopia: a Spatio-temporal analysis from 2005 to 2019

**DOI:** 10.1186/s12913-022-08850-1

**Published:** 2022-11-30

**Authors:** Binyam Tariku Seboka, Tizalegn Tesfaye Mamo, Tensae Mekonnen

**Affiliations:** 1 School of Public Health, Dilla University, Dilla, Ethiopia; 2 Translational Health Research Institute (THRI), School of Medicine, Western Sydney University, Penrith, NSW 2751, Australia

**Keywords:** Ethiopian demographic and Health Survey (EDHS), Maternal care utilization, Antenatal care (ANC), Birth attended in a health facility, ANC utilization, Spatial analysis, Geographically weighted regression (GWR)

## Abstract

**Introduction:**

Inequalities in maternal care utilization pose a significant threat to maternal health programs. This study aimed to describe and explain the spatial variation in maternal care utilization among pregnant women in Ethiopia. Accordingly, this study focuses on identifying hotspots of underutilization and mapping maternal care utilization, as well as identifying predictors of spatial clustering in maternal care utilization.

**Methods:**

We evaluated three key indicators of maternal care utilization: pregnant women who received no antenatal care (ANC) service from a skilled provider, utilization of four or more ANC visits, and births attended in a health facility, based the Ethiopian National Demographic and Health Survey (EDHS5) to 2019. Spatial autocorrelation analysis was used to measure whether maternal care utilization was dispersed, clustered, or randomly distributed in the study area. Getis-Ord Gi statistics examined how Spatio-temporal variations differed through the study location and ordinary Kriging interpolation predicted maternal care utilization in the unsampled areas. Ordinary least squares (OLS) regression was used to identify predictors of geographic variation, and geographically weighted regression (GWR) examined the spatial variability relationships between maternal care utilization and selected predictors.

**Result:**

A total of 26,702 pregnant women were included, maternal care utilization varies geographically across surveys. Overall, statistically significant low maternal care utilization hotspots were identified in the Somali region. Low hotspot areas were also identified in northern Ethiopia, stretching into the Amhara, Afar, and Beneshangul-Gumuz regions; and the southern part of Ethiopia and the Gambella region. Spatial regression analysis revealed that geographical variations in maternal care utilization indicators were commonly explained by the number of under-five children, the wealth index, and media access. In addition, the mother’s educational status significantly explained pregnant women, received no ANC service and utilized ANC service four or more times. Whereas, the age of a mother at first birth was a spatial predictor of pregnant who received no ANC service from a skilled provider.

**Conclusion:**

In Ethiopia, it is vital to plan to combat maternal care inequalities in a manner suitable for the district-specific variations. Predictors of geographical variation identified during spatial regression analysis can inform efforts to achieve geographical equity in maternal care utilization.

**Supplementary Information:**

The online version contains supplementary material available at 10.1186/s12913-022-08850-1.

## Introduction

Maternal health refers to women’s health during pregnancy, childbirth, and the postnatal period [1–3]. Improving the adherence to and utilization of proper care during pregnancy and childbirth has been an essential way of reducing the risk of maternal and child deaths. Globally, maternal health programs have greatly expanded, and maternal mortality has been reduced substantially over the past two decades [4–6]. Despite the progress in reducing maternal deaths, more than 295,000 women died during and following pregnancy and childbirth in 2017 [4, 7]. The majority of the maternal mortality rate (MMR) is observed in developing countries, with 66% of the maternal deaths reported in sub-Saharan Africa and central and southern Asia in 2020 [4, 8]. Many developing countries’ massive burden of these deaths resulted from complications related to pregnancy and childbirth [7–9].

In sub-Saharan Africa, the MMR is estimated to reach more than 542 per 100,000 mothers during and following pregnancy and childbirth [4, 6]. The maternal mortality rate in Ethiopia is 67 deaths per 1000 live births [10], with 2500 maternal deaths in 2020 [5], making it one of the highest in sub-Saharan Africa. There was a reduction of 50% in maternal death rates between 2000 and 2020 [11]. Still, MMR remains unacceptably high to achieve Ethiopia’s goal of ending preventable maternal mortality and achieving Sustainable Development Goals (SDGs) [12, 13]. In 2015, the World Health Assembly approved a transition in strategy from ‘ending preventable maternal mortality (EPMM)” to “reducing global MMR to less than 70 per 100 000 live births by 2030,“ with a major transformation in national maternal and child health efforts [9]. However, a gap in the implementation, integration, and scale-up of maternal health services has been widely observed and is a major threat to these programs [14, 15]. To close these gaps and reduce maternal mortality, strengthening and scaling up maternal health services are necessary. Thus, to ensure that all women have access to respectful and high-quality maternity care, it is critical to address inequities that affect maternal health outcomes, particularly since they affect women’s rights [1, 8, 16, 17].

Evidence shows that precise mapping and modeling of maternal-health-related services [18, 19], including maternal care utilization during pregnancy and childbirth, provide in-depth analysis to prevent maternal deaths. Previous studies on pregnant women’s utilization of ANC care [20–27], utilizing ANC service four or more times [21, 24, 25, 28, 29], and birth attended in a health facility [30–35] have found significant geographical variations across these indicators. Though, there has been a limited attempt to quantify the geographical heterogeneity of maternal care utilization indicators among Ethiopian regions. Furthermore, there was limited evidence of factors associated with the geographic clustering of these services.

Hence, this study aimed to describe and explain spatial variation in maternal care utilization among pregnant women in Ethiopia, based on nationally representative data obtained from four previous Ethiopian demographic health surveys (EDHS). Accordingly, this study focuses on identifying hotspots of underutilization and mapping maternal care utilization, as well as identifying predictors of spatial clustering in maternal care utilization. Findings would be essential to provide geographic-based evidence for decision-makers and planners to formulate strategies and act appropriately, aiming to reduce inequalities in maternal care services in Ethiopia.

## Methods

### Study design and setting

We used four data sets from the EDHS surveys conducted in 2005, 2011, 2016, and 2019 [36–39]. Ethiopia is located in the horn of Africa and has a population of 110,613,986 people [40–42], making it the second-most populous country in Africa. The country has nine regional states and two city administrations, with each region subdivided into zones, districts (Woreda), towns, and kebeles (the smallest administrative units) [43–45].

The Demographic and Health Survey (DHS) program is an international effort designed to collect accurate, nationally representative data on health and population in developing countries through household surveys [36–39]. A stratified two-stage cluster sampling procedure was employed for all surveys. The DHS adopted a multistage sampling approach involving the selection of clusters in the first stage and the subsequent selection of households in the second stage. Cluster selection was also stratified by place of residence (i.e., rural/urban) and districts (Woreda). Each woreda is further subdivided into kebeles, and each kebele is also subdivided into census enumeration areas (EAs). Spatial information or location data (latitude and longitude coordinates) were also taken from selected enumeration areas. The study design and setting are detailed elsewhere the Central Statistical Agency (CSA) Ethiopia report [36–39]. The district demarcation shapefile for Ethiopia was obtained from the CSA (Central Statistical Agency) database.

### Source and study population

The source population was all reproductive-age women (15–49 years old) across Ethiopia, whereas the study population consisted of reproductive-age women who had had their last delivery in the five years preceding the survey. Our choice of the most recent birth is based on the assumption that information on maternal health care for the most recent birth is less subject to recall bias. Accordingly, 55,153 women aged 15–49 were surveyed from 2005 to 2019 for the EDHS, and 26,702 women who had had their last delivery in the five years preceding the survey were considered in the analysis. The classification was informed by literature that used the DHS data sets, and the detailed sampling procedure was presented in the full DHS report [36–39].

### Study tool and measurement

#### Outcome variables of the study

We constructed the three maternal care indicators according to the DHS maternal care definitions. The outcome variables are: no antenatal care (ANC) service from a skilled provider, utilization of four or more antenatal care (ANC) visits, and birth attended in a health facility [36–39]. In the DHS, mothers were asked whether they had obtained ANC service during the pregnancy for their most recent live birth in the five years preceding the survey. Pregnant women who received no ANC service from a skilled provider were represented when women did not receive ANC-related health services. For utilization of four or more ANC visits for their most recent live birth, the resulting score of the pregnant women’s number of visits was dichotomized as 0 and 1, with 0 referring to “women who had three or less than three ANC visits” and 1 referring to “women who had four or more ANC visits.“ Furthermore, for each live birth over the same period, mothers were also asked what type of assistance they received at the delivery time. Accordingly, we created the use of “birth attended in a health facility during delivery” to indicate whether or not a woman used a health facility during delivery.

#### Explanatory variables

The selection of the explanatory variables included in this paper was based on literature about maternal care utilization [19–21, 24, 26, 28, 30, 31, 34, 46–48]. The explanatory variables used in the study were; mothers’ age, marital status, level of education, media exposure, wealth status, owning a mobile phone, radio, or television set in the household, participation in women’s development groups, number of pregnancies, births, history of abortion, history of stillbirth, unplanned pregnancy, knowledge about ANC benefits, and women’s household decision-making capacity. Women’s household decision-making capacity was derived from three questions: “decisions on personal health care,“ “decisions on large household purchases,“ and “decisions on visits to family or relatives.“ These response categories were recoded as “not alone = 0” and “alone = 1”). An index was created with all the “yes” and “no” answers, with scores ranging from 0 to 3. The scores 0 and 1 were labeled as “less capacity,“ whereas 2 and 3 were labeled as “more capacity.“

Then the considered variables were assessed for their random geographical distribution across the study areas before using them in the spatial analysis. Finally, selected variables in modeling spatial relationships at the household level were the proportions of women owning mobile phones, radios, television sets, or access to media in the household, the household wealth index, the region, and the residence. At the individual level, proportions of the education status of the mother, the age of the mother, and the number of pregnancies and births were considered.

### Data management and statistical analysis

Before conducting spatial analysis, STATA version 14.2 software was used to maintain the representativeness of sampled maternal records; we used sampling weights and a stratified sample design to produce estimates. Furthermore, the weighted proportions of maternal care utilization indicators and explanatory variables were performed in STATA. The variables were exported to Geographical Information System (ArcGIS version 10.8) software for visualization, exploration, and spatial modeling analysis.

#### Spatial autocorrelation analysis

The spatial autocorrelation or Global Moran’s I statistic test was used to measure whether patterns of maternal care utilization indicators, which include pregnant women who received no ANC service from a skilled provider, utilization of four or more ANC visits, and births attended in a health facility, were randomly distributed, dispersed, or clustered in Ethiopia [36–39]. The calculated Moran’s I values close to − 1 indicate maternal care utilization is dispersed. In contrast, an I value close to + 1 indicates that maternal care utilization is clustered, and if the I value is zero, the maternal care utilization is distributed randomly [49, 50]. Furthermore, a statistically significant Moran’s I (p 0.05) leads to the rejection of the null hypothesis (random distribution of the maternal care utilization among pregnant women who gave birth during the past five years of each survey) and indicates the presence of spatial autocorrelation [50, 51].

#### Hot spot analysis (Getis-Ord Gi* statistic)

Hot Spot Analysis or Getis-Ord Gi* statistics were employed to measure how spatial-autocorrelation differs by study location by calculating Gi* statistics for each area. The Z-score was calculated to ensure the statistical significance of maternal care utilization clustering, and the p-value calculated the significance *p*value *<* 0.05 at 95% CI. If the Z-score is between 1.96 and + 1.96, the *p*-value must be greater than 0.05, and vice versa. If the *p*-value is less than-1.96, it is declared a cold spot, and if greater than + 1.96, it is declared a hotspot area [52].

#### Spatial interpolation

The spatial interpolation technique was employed to predict maternal care utilization in the un-sampled areas of the country based on the sampled enumeration areas. There are various deterministic and geostatistical interpolation methods. In this study, ordinary Kriging is used since it incorporates spatial autocorrelation and statistically optimizes the weight. Accordingly, the Kriging spatial interpolation method was used to estimate the geographical distribution of inequalities in maternal care in unsampled areas [53].

#### Spatial regression (modeling spatial relationships)

After detecting the hot spot areas of maternal care utilization, spatial regression modeling was employed to identify predictors of the observed spatial clustering of maternal care utilization: pregnant women who received no ANC service from a skilled provider, utilization of four or more ANC visits, and births attended in a health facility, using 2019 survey data.

To further untangle the spatial relationship between explanatory variables and maternal care utilization indicators, we have employed both global and local regression modeling approaches. On a global scale, we used the global OLS linear regression model. Spatial autocorrelation in the residuals of the OLS models was then again tested by Moran’s I statistics to ascertain that the residual was not clustered. While the joint Wald statistic indicated the overall model significance (p *<* 0.01), the robust probabilities showed coefficient significance (p *<* 0.01) for the explanatory variables. However, simple linear regression assumes changes across a study area to be universal; variations across geographical space might be lost. In addition, a particular independent variable may be a strong predictor in one cluster but not in another. As a result, at the local scale, we have employed GWR, which calibrates a regression model on each spatial unit of analysis and weights different regression parameters anywhere in the study area given a response and a set of predictor variables.

In this study, we have used the same set of variables included in the OLS model to specify GWR models to account for spatial nonstationarity in the relationships between the maternal care utilization indicators and predictor variables. Then, the coefficients that were created using GWR were mapped. A positive coefficient means that predictors and maternal care utilization indicators changed in the same direction, and a negative coefficient indicates the existence of a vice-versa relationship [51, 52]. Furthermore, the OLS and GWR models were compared using different parameters.

## Ethical considerations

We made the request, and permission was granted to download and utilize the data for this study from the DHS program, http://www.dhsprogram.com; the data is freely accessible to anyone. All data obtained from the DHS is de-identified data before sharing it with the public. Thus participants’ identifiers were removed, and institutional ethical review was waived, ensuring the regulations for the protection of human subjects.

## Results

### Trends of maternal care utilization in Ethiopia

In this study, data on 26,702 women who gave birth during the past five years of each survey were included in the analysis from four consecutive surveys conducted in 2005, 2011, 2016, and 2019. Table 1 describes the magnitude of maternal care utilization among pregnant women in Ethiopia.

**Table 1:** Trends of maternal care utilization among pregnant women in Ethiopia (2005–2019)

	**Pregnant who received no ANC service from a skilled provider (%)**					**Utilization of four or more ANC visit (%)**					**Birth attended in a health facility (%)**				
	**EDHS**					**EDHS**					**EDHS**				
	**2005**	**2011**	**2016**	**2019**	**Pooled**	**2005**	**2011**	**2016**	**2019**	**Pooled**	**2005**	**2011**	**2016**	**2019**	**Pooled**
**Regions**															
**Tigray**	62.5	35	10	6	28.3	17.9	30.7	56.5	63.9	42.25	6.1	11.6	56.9	72.4	36.7
**Afar**	83	64.7	48.7	37.4	58.4	8	11.4	20.6	31.1	17.7	3.9	6.8	14.7	28.3	13.4
**Amhara**	73.1	59.1	32.9	17.9	45.7	7.3	12.4	31.5	50.8	25.5	3.5	10.2	27.1	54.2	23.7
**Oromia**	74.5	60.5	49.3	29.2	53.3	10.1	18.6	22.1	40.6	22.8	4.2	8	18.8	40.9	17.9
**Somali**	92	74.7	56.4	69.8	73.2	4.4	7	11.8	11.2	8.6	5	7.6	17.9	23.3	13.4
**Benishangul**	74.3	59	31.3	16.7	45.3	10.6	15.9	42	55.9	31.1	4.6	9.1	25.7	63.6	25.7
**SNNPR**	68.5	59.2	30.2	30.6	47.1	15	17.7	38.2	34.1	26.2	3.7	6.2	25.5	47.6	20.7
**Gambella**	61	42.3	27.7	14.3	36.3	26.6	30.8	43.5	31.8	33.1	15.2	27.5	45	70.3	39.5
**Harari**	58	40.5	24.1	19.3	35.4	25.9	35	34.9	38.8	33.6	31.6	32.4	50.2	64	44.5
**Addis Abeba**	11.5	5.4	3.2	3.1	5.8	80.6	86.4	89.1	81.8	84.4	78.5	82.3	96.6	94.8	88.0
**Dire Dawa**	45.7	38.7	12.6	16.2	28.3	34.6	38.9	66	61.7	50.3	25.8	39.7	56.2	70.7	48.1
**Total **	64.0	49.0	29.6	23.6	41.5	21.9	27.7	41.4	45.6	34.1	16.5	21.9	39.5	57.2	33.8

Across all surveys, the overall proportion of maternal care utilization among pregnant women has improved. The pooled estimate of pregnant women who received no ANC service from a skilled provider in Ethiopia was 41.5%, but it has declined from 64.0% to 2005 to 23.6% in 2019. The estimated proportion of pregnant women who received no ANC service from a skilled provider shows regional variations consistently over time. In the 2019 EDHS, the highest proportion was in Somali regions with 69.8%, while only 3.1% was in Addis Ababa. Furthermore, the pooled estimate of utilizing ANC four or more times was 34.1%. Similarly, the utilization pattern of four or more ANC visits shows that pregnant women from Addis Ababa had a better level of utilization compared to other regions. Regarding births attended in a health facility, the proportion of women who gave birth at the facility significantly increased from 16.5% to 2005 to 57.2% in 2019, as can be seen in Table 1. Likewise, the magnitude of health facility delivery shows variation across regions.

### Spatial variation of maternal care utilization in Ethiopia

Across four surveys (2005–2019), the Global Moran’s I values revealed a significantly clustered pattern for each maternal care utilization indicator at a 0.01 level of significance (See Supplementary File 1: spatial autocorrelation report). This indicates the need to examine each indicator across regions.

#### Hotspot analysis of maternal care utilization in Ethiopia

**Fig. 1:**
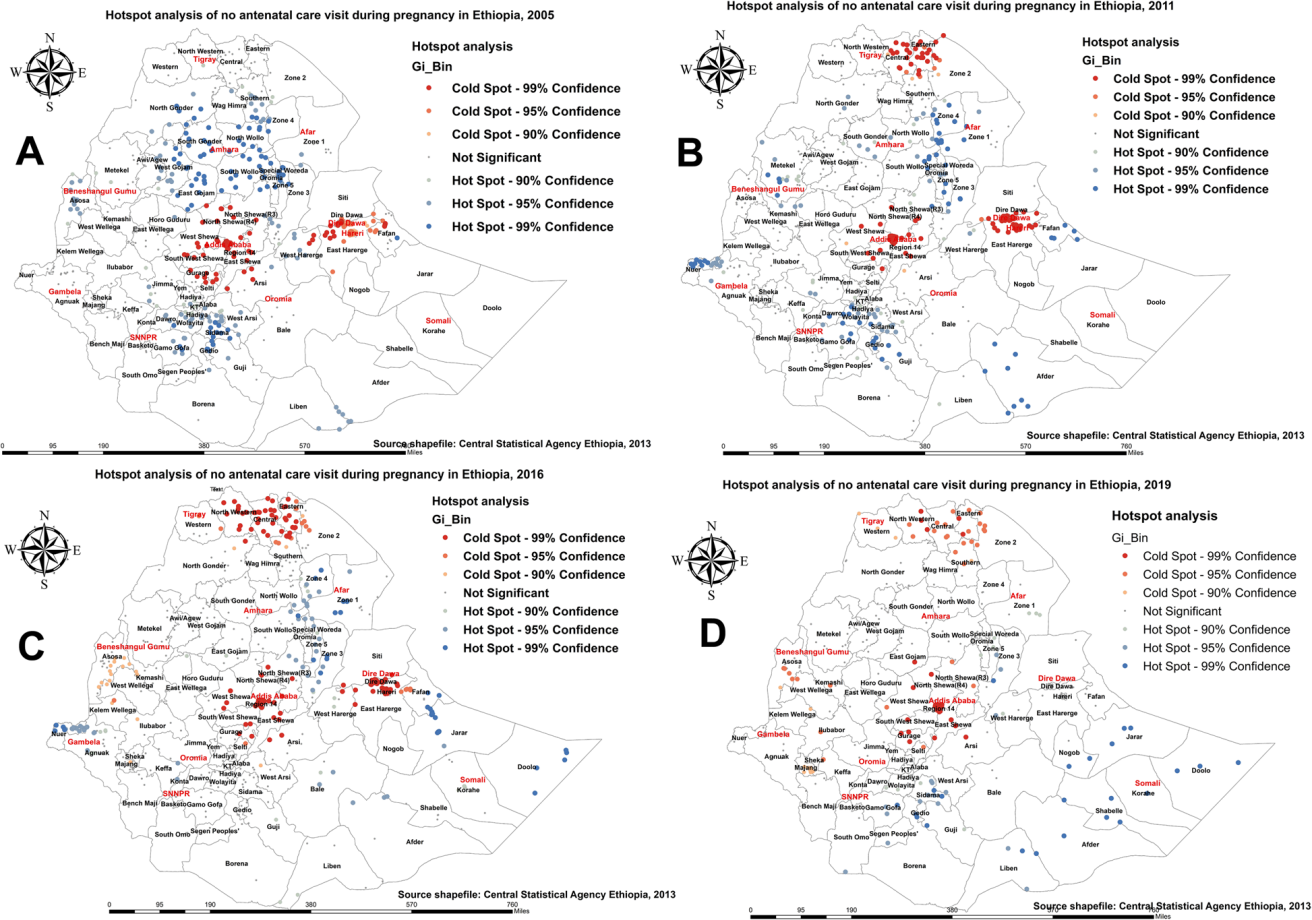
Hotspot analysis of pregnant who received no antenatal care(ANC) service from a skilled provider in Ethiopia: 2005 (**A**), 2011 (**B**), 2016 (**C**), and 2019 (**D**)

Regarding pregnant women who received no ANC service from a skilled provider, in the 2005 EDHS (Fig. 1A), hotspots of pregnant women who received no ANC service were identified in the districts of the northern, southern, and eastern parts of Ethiopia (Western Tigray; Wag Himra, South Gondar; and Oromia zone of the Amhara region; Zones 2 and 4 of Afar; Illubabor, North Shewa, and Borena of Oromia; and Liben, Afder, and Siti of the Somali region; also districts in Beneshagual Gumz; and districts in the Beneshagual Gumz; also districts in Beneshagual Gumz; Liben In EDHS-2011 (Fig. 1B), similar patterns were observed: hotspot areas were reduced in districts of northern Ethiopia, and districts were stretched in the Gambella and Somali regions. In the 2016 EDHS (Fig. 1C), the highest hotspots were observed in the districts of Afar, Gambella, Somali, and a few districts of the Amhara region. In the 2019 EDHS, the highest hotspots were observed in most districts of the Somali region, followed by a few districts in the SNNP and Southern Oromia regions (Fig. 1D). Overall, we noted some variations over the last two decades. For example, compared to pregnant women surveyed in 2005 (Fig. 1A), the hotspots of pregnant women who received no ANC service from a skilled provider were significantly reduced in 2019 (Fig. 1D). Over the last two decades, hotspot areas for pregnant women who received no ANC service from a skilled provider were consistently observed in the districts of the Somali region.

**Fig. 2:**
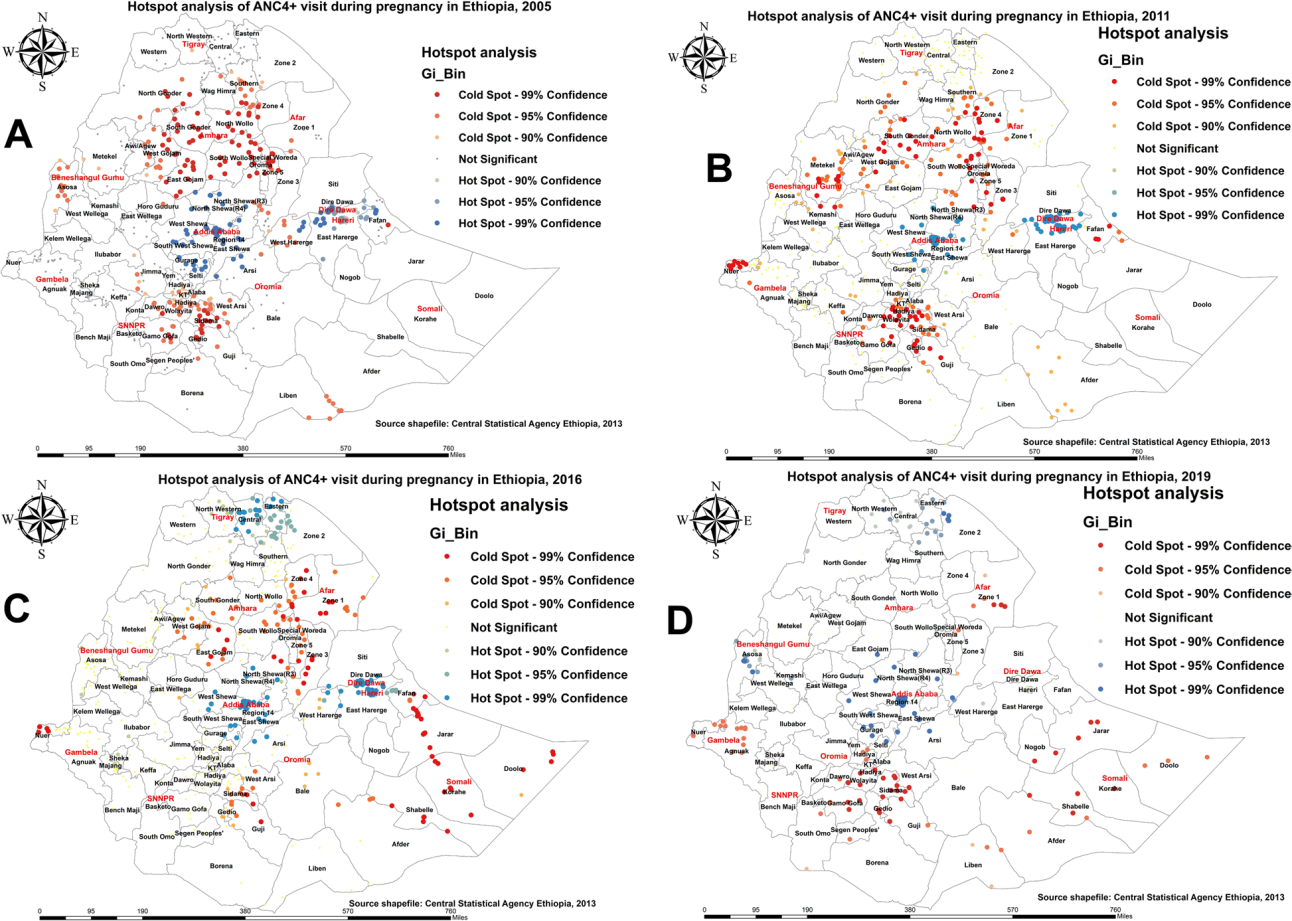
Hotspot analysis of utilization of four or more antenatal care (ANC) visit among pregnant women in Ethiopia: 2005 (**A**), 2011 (**B**), 2016 (**C**), and 2019 (**D**)

Figure 2 shows hotspot and coldspot areas with a high and low proportion of pregnant women who utilized ANC services four or more times at the district level. As seen in Fig. 2A and B, in 2005 and 2011, the districts with low hotspots of utilizing ANC four or more times in the Amhara region are North Wollo, South Wollo, South Gondar, West Gojam, East Gojam, North Shewa, Wag Hemira, and the Oromia zone. The zone identified in the Tigray region is found in the southern and central parts. Similarly, four zones from the Afar region, three from the Somali region, and the Nuer and Anuak districts in the Benishangul-Gumuz region were consistently associated with lower proportions of pregnant women’s utilization of four or more ANC visits. In the 2016 and 2019 EDHS, the lowest proportion in the utilization of four or more ANC visits was identified in a few parts of northern and southern Ethiopia and most districts of the Somali region (Fig. 2C, D). On the other hand, the spatial clustering of high hotspots of pregnant women who utilized ANC services four or more times was consistently observed in the central part of Ethiopia (Fig. 2).

**Fig. 3:**
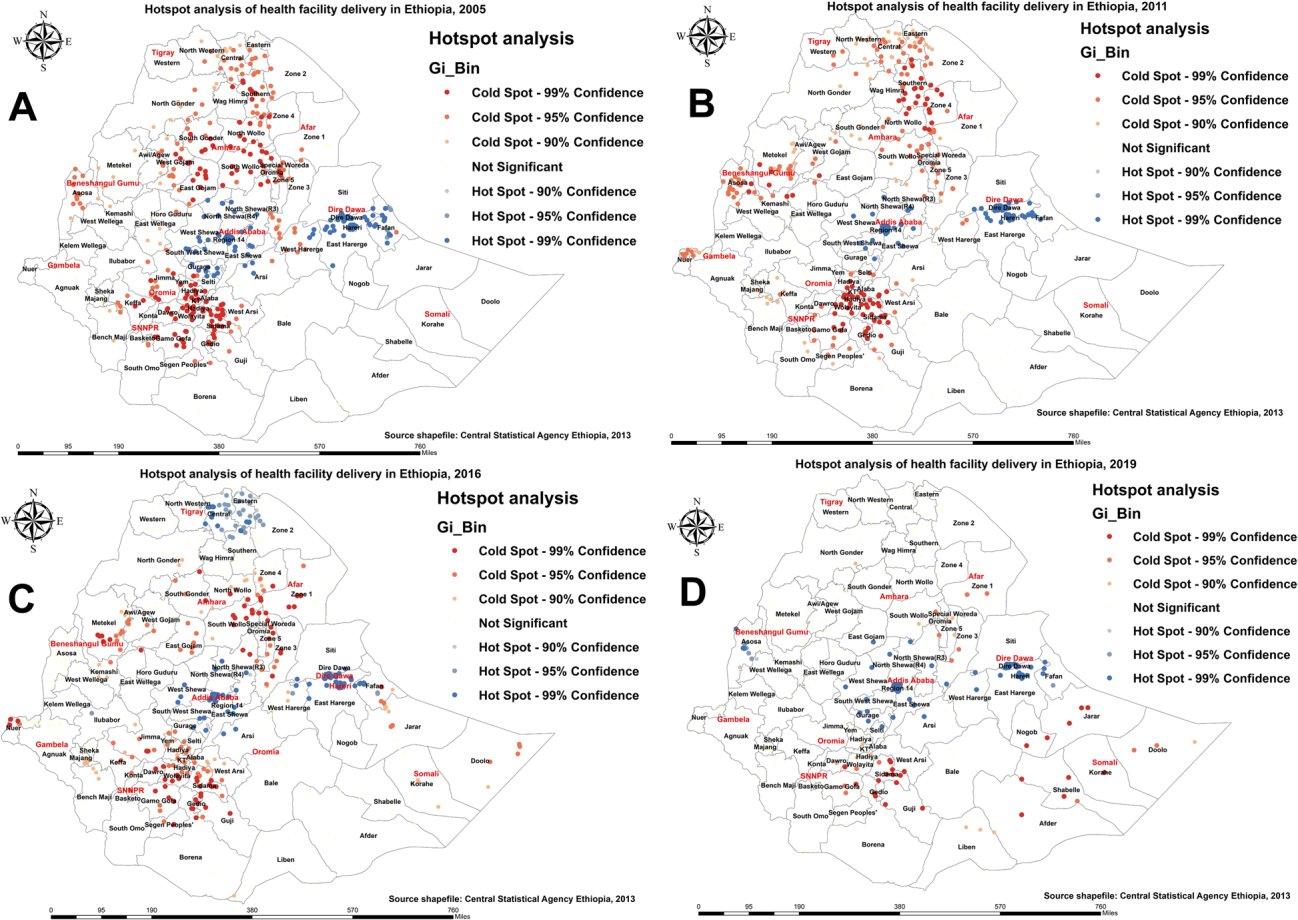
Hotspot analysis of birth attended in a health facility in Ethiopia: 2005 (**A**), 2011 (**B**), 2016 (**C**), and 2019 (**D**)

Figure 3 shows hotspot and coldspot areas with a high and low proportion of births attended in the health facility at the district level. As shown in Fig. 3A, B, in the 2005 and 2011 EDHS, districts from West Gojjam, South Wollo, and South Gondar of the Amhara region; southern parts of the Tigray region; Gurage, Woliyta, Sidama, Kaffa, and Gamo Gofa of the SNNPR region; Jima in the Oromia region; and Metekel and Kamashe of the Benishangul-Gumuz region were significantly associated with low births attended in a health facility, while districts from the central and Harari and Dire Dawa parts of eastern Ethiopia were significantly associated with high births attended in a health facility. In the 2016 and 2019 EDHS, the lowest number of births attended in a health facility was identified in a few parts of the Amhara, SNNPR, and Gambella regions and most districts of the Somali region (Fig. 3C, D). We observed that the proportion of low births attended in a health facility is worse in the Somali region of Ethiopia.

#### Kringing interpolation of maternal care utilization in Ethiopia

**Fig. 4:**
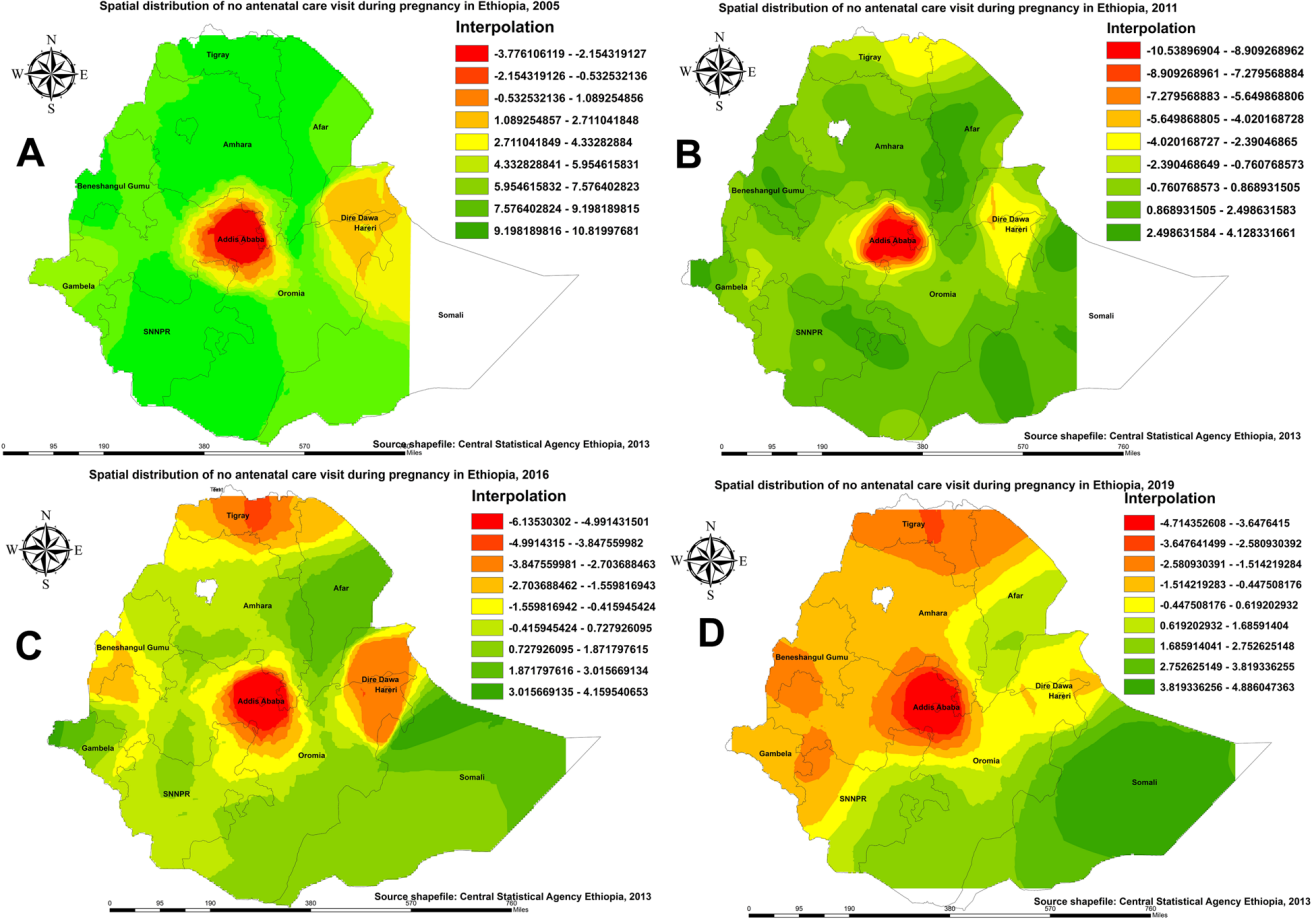
Spatial Interpolation of pregnant who received no antenatal care(ANC) service from a skilled provider in Ethiopia: 2005 (**A**), 2011 (**B**), 2016 (**C**), and 2019 (**D**)

The green color indicates predicted areas with a higher proportion of pregnant women who received no ANC service from a skilled provider, and the red color indicates areas with a lower proportion of pregnant women who received no ANC service from a skilled provider (Fig. 4). Based on EDHS 2005, Kriging interpolation predicts that a higher proportion of pregnant women received no ANC service from a skilled provider in the Amhara, Tigray, border areas of Afar, SNNPR, Benishangul-Gumuz, and the southern part of Oromia regions, whereas utilization of ANC service during pregnancy was predicted in the central (Addis Ababa, North-Shewa of Oromia), eastern (Harari, and Dire Dawa) parts of Ethiopia (Fig. 4A). In the 2011 EDHS, some parts of Amhara, Afar, SNNPR, Benishangul-Gumuz, Somali, and Gambella regions were predicted to be areas more prevalent for pregnant women who received no ANC service from a skilled provider compared to other regions (Fig. 4B). In 2016, parts of the Somali and Gambella regions were predicted to have more pregnant women who received no ANC service from a skilled provider (Fig. 4C). In the 2019 survey, most areas in the Somali region were predicted to have pregnant women who received no ANC service from a skilled provider (Fig. 4D).

**Fig. 5:**
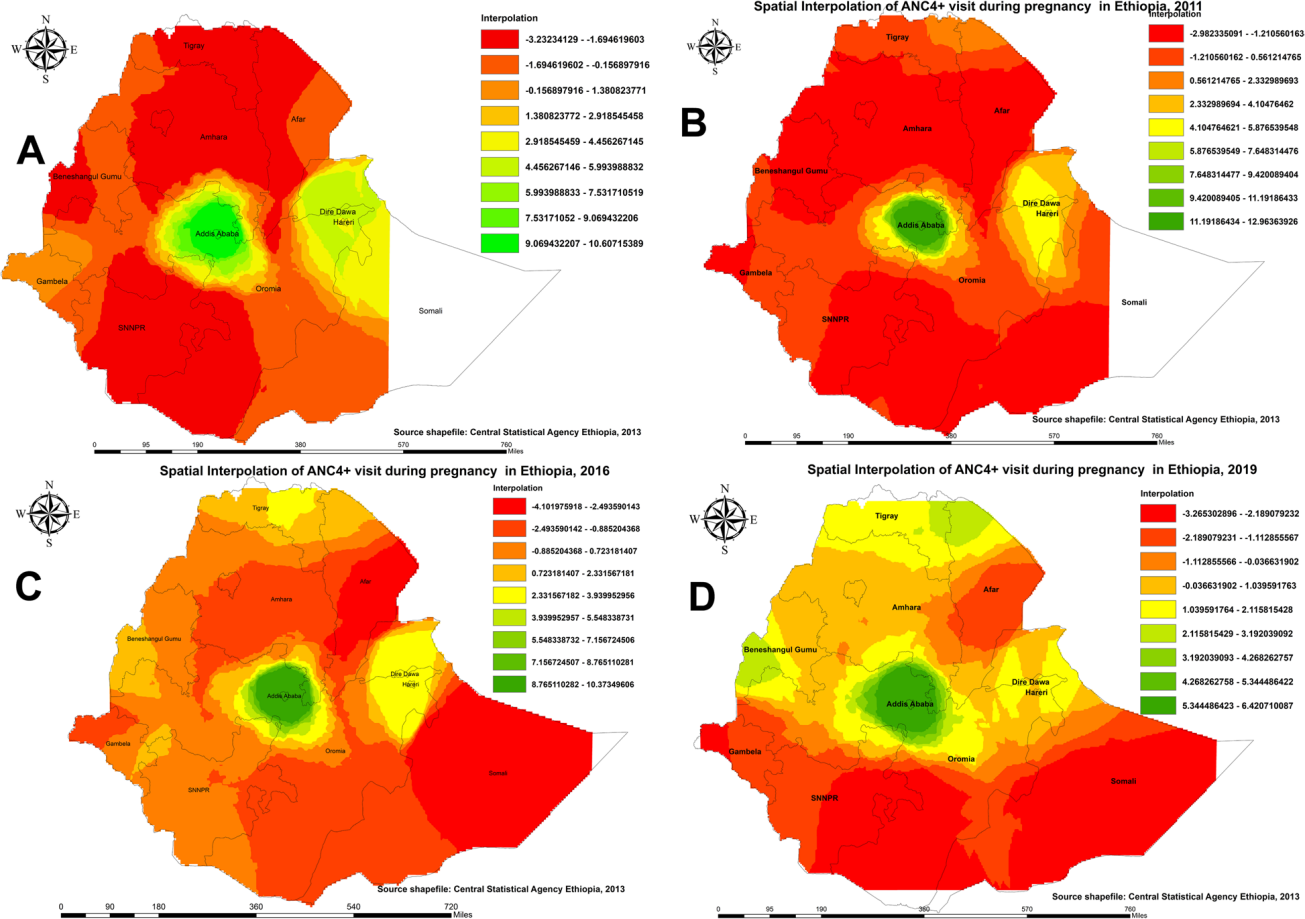
Spatial Interpolation of utilization of four or more antenatal care (ANC) visit among pregnant women in Ethiopia: 2005 (**A**), 2011 (**B**), 2016 (**C**), and 2019 (**D**)

Regarding spatial prediction in the utilization of four or more ANC visits, in the 2005 and 2011 EDHS, most parts of regions in Ethiopia were predicted as a more typical area of low utilization of four or more ANC visits as compared with the central and eastern (Harar and Dire Dawa) parts of the country (Fig. 5A, B). In the 2016 and 2019 EDHS surveys, significant improvements were observed in the utilization of four or more ANC services among pregnant women (Fig. 5C, D).

**Fig. 6:**
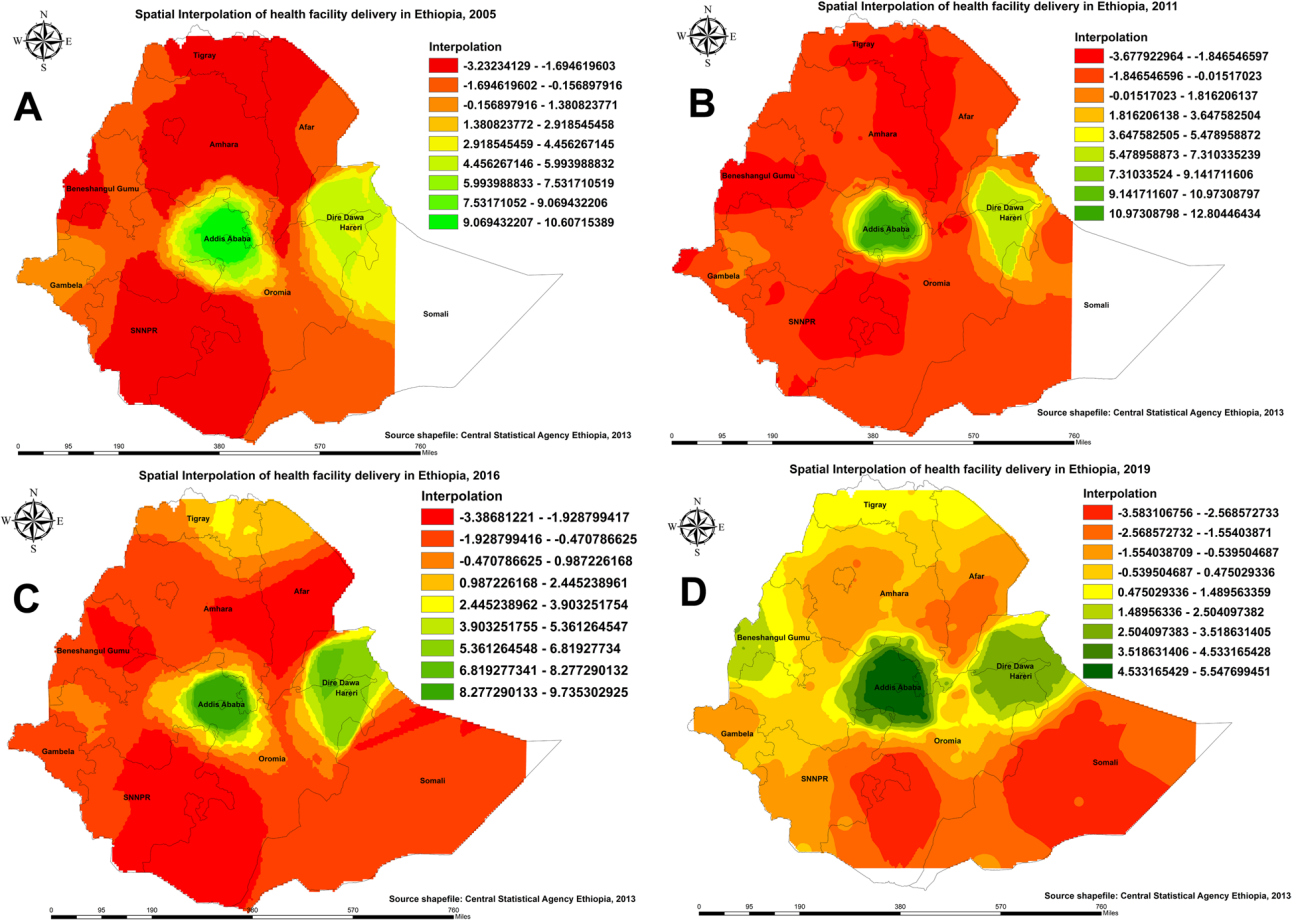
Spatial Interpolation of birth attended in a health facility in Ethiopia: 2005 (**A**), 2011 (**B**), 2016 (**C**), and 2019 (**D**)

Figure 6 shows predicted areas of high and low rates of birth attended in a health facility in Ethiopia. As can be seen in Fig. 5A and B in the 2005 and 2011 surveys, most parts of central Ethiopia had a higher proportion of births attended in a health facility, with a significant stretch in Addis Ababa and eastern parts of Harar and Dire Dawa. In 2016 (Fig. 4C), Kriging interpolation revealed that the highest predicted proportion of births attended in a health facility was stretched in parts of northern Ethiopia, such as the Tigray region. As illustrated in Fig. 4D for 2019, Kriging interpolation predicts that the proportion of births attended in a health facility has significantly improved throughout the country. However, the spatial clustering of low births attended in a health facility was consistently observed in the Somali region and the southern part of Ethiopia.

### Spatial regression of maternal care utilization in Ethiopia

In this study, an OLS and GWR analysis was carried out on each maternal care utilization indicator. As illustrated in Table 2, the OLS regression model explained about 53.0%, 52.0%, and 63.0% of the variation in pregnant women who received no ANC service from a skilled provider, utilized four or more ANC visits, and birth in a health facility, respectively. Regarding their performance, we compare the OLS and GWR models using their Akaike Information Criterion (AICc), multiple, and adjusted R-squared values. It can be stated that the GWR model is a better fit model.

**Table 2:** Diagnostics summary result of OLS and GWR models of maternal care utilization indicators

	**Diagnostic criteria**	**Maternal care utilization indicators**					
		**Pregnant who received no ANC service from a skilled provider**		**Utilization of four or more ANC visit**		**Birth attended in a health facility**	
		**Magnitude**	***p*-value**	**Magnitude**	***p*-value**	**Magnitude**	***p*-value**
	**Number of observation**	305		305		305	
**OLS**	**AICs**	-177.348		-108.18		-70.754	
	**R squared**	0.545		0.533		0.631	
	**Adjusted R squared**	0.536		0.522		0.624	
	**Joint F Statistics**	59.577	0.000	84.002	0.000	169.49	0.000
	**Joint Wald Statistic**	331.312	0.000	458.77	0.000	890.987	0.000
	**Koenker (BP) Statistic**	54.830	0.000	6.900	0.141	13.968	0.003
	**Jarque-Bera Statistic**	3.288	0.193	2.632	0.268	10.281	0.006
**GWR**	AICc	-250.450		-160.812		-91.234	
	Multiple R2	0.685		0.648		0.673	
	Adjusted R2	0.647		0.611		0.654	

#### Factors associated with the spatial variation of pregnant who received no ANC service from a skilled provider

The findings of GWR indicated that household wealth index, access to TV, educational level, number of under-five children, and age of a mother at first birth were predictors that significantly explained the spatial dependence of pregnant women who received no ANC service from a skilled provider (Table 3).

**Table 3:** Spatial regression summary result of OLS (global GWR) coefficients for maternal care utilization indicators in Ethiopia

Variable	Coefficient	Standard error	t-statistic	Probability	Robust probability	VIF
**Pregnant who received no ANC service from a skilled provider**						
Intercept	-0.022	0.091	-0.239	0.811	0.808	
Household wealth index (poor)	-0.225	0.047	-4.785	0.000	0.000	2.962
Women radio(no radio)	0.125	0.064	1.961	0.050	0.028	1.742
No TV access (no access)	-0.003	-0.050	-0.070	0.943	0.936	3.238
No education	0.335	0.062	5.353	0.000	0.000	2.368
More than 2 under-five	0.241	0.054	4.444	0.000	0.000	1.407
Above 20 age	0.223	0.082	2.709	0.007	0.008	1.719
**Utilization of four or more ANC visit**						
Intercept	0.422	0.057	7.371	0.000	0.000	
Household wealth index (poor)	0.133	0.055	2.405	0.016	0.0124	3.244
Women education (had formal education)	-0.120	0.069	-1.739	0.083	0.089	2.279
Media access (had access)	0.327	0.061	5.353	0.000	0.000	2.930
Number of under-five children (had one child)	-0.311	0.060	-5.167	0.000	0.000	1.377
**Birth attended in a health facility**						
Intercept	0.308	0.043	7.144	0.000	0.000	
Household wealth index (poor)	0.333	0.056	5.885	0.000	0.000	3.001
Media access (had access)	0.409	0.062	6.618	0.000	0.000	2.634
Number of under-five children (more than 2)	-0.286	0.062	-4.585	0.000	0.000	1.297

**Fig. 7:**
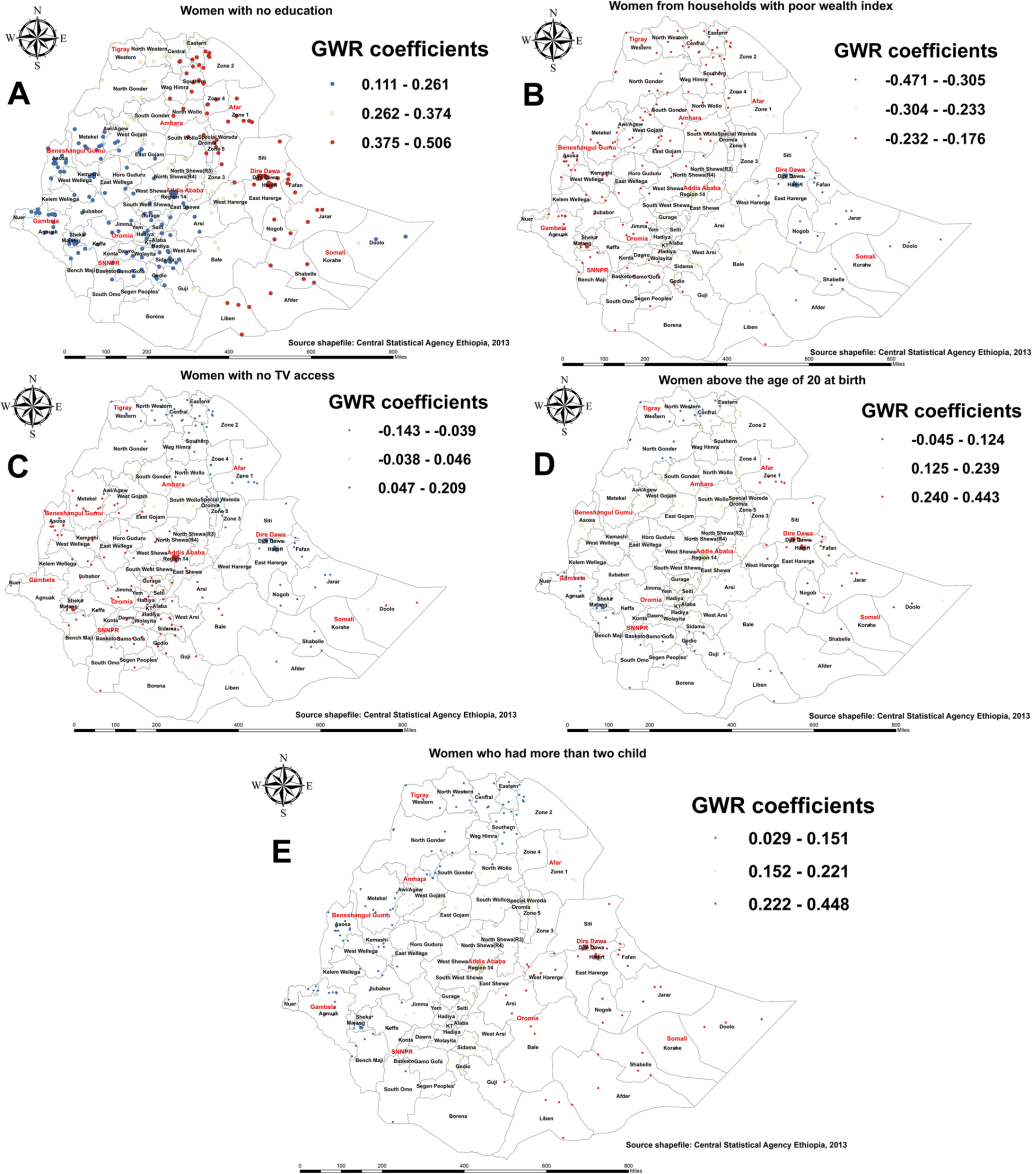
Geographically weighted regression coefficient estimates for predictors of pregnant who received no antenatal care(ANC) service from a skilled provider in Ethiopia

Lack of education was predominantly positively associated with pregnant women who did not receive ANC service from a skilled provider, with the coefficient ranging from 0.111 to 0.506. As shown in panel A of Fig. 7, when the proportion of uneducated women increases, the proportion of pregnant women who received no ANC service from a skilled provider also increases in the eastern, southern, and central parts of Tigray, some parts of the Amhara region (mainly in north Wollo, Waghmera, and Oromia zones), and in parts of the Afar, Dire Dawa, Harari, and Somali regions of Ethiopia. Similarly, having under-five children in the household also shows a positive association, with the coefficient ranging from 0.029 to 0.448; it was a strong predictor in parts of the Oromia, Dire Dawa, Harari, and Somali regions (Fig. 7E). Panel D of Fig. 7 indicates a positive association between women above 20 years of age at their first birth, mainly in Zone 1 of Afar, west Hararge of Oromia, and parts of Harari, Dire Dawa, and the Somali region, and pregnant women who received no ANC service from a skilled provider. In addition, lack of media access shows a positive association in southern and western parts of Ethiopia (Fig. 7C).

This study also highlights the space-dependent relationship between pregnant women who received no ANC service from a skilled provider and wealth, with the coefficient ranging from − 0.471 to -0.176. As shown in panel B of Fig. 7, poor households in most parts of Ethiopia (Amhara, Tigray, Benishangul-Gumuz, Gambella, SNNPR, western and central parts of Oromia), and Liben Zone in the Somali region, were negatively associated with pregnant women who received no ANC service from a skilled provider.

#### Factors associated with the spatial variation of the utilization of four or more ANC visits

GWR analysis revealed education, the household wealth index, the number of under-five children in the household, and access to the media significantly explained the spatial dependence of pregnant women’s utilization of ANC services four or more times (Table 3).

**Fig. 8:**
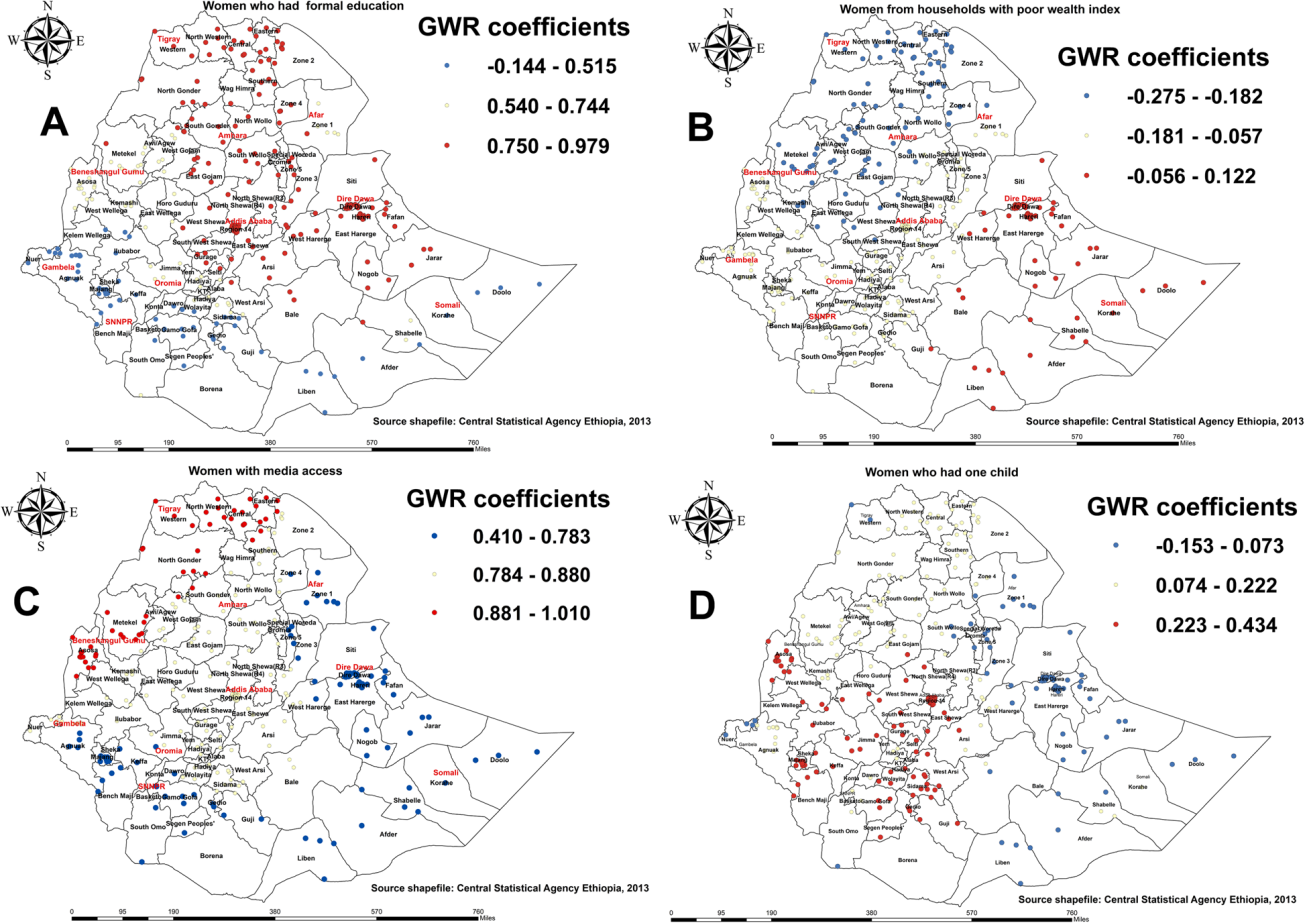
Geographically weighted regression coefficient estimates for predictors of utilization of four or more antenatal care (ANC) visit among pregnant women in Ethiopia

As shown in Fig. 8A, formal education was a strong predictor of utilizing ANC services four or more times, with the coefficients ranging from 0.750 to 0.979. As the proportion of women with formal education increases, the utilization of four or more ANC visits increases in parts of the Amhara region, Tigray, Metekel of Benishangul-Gumuz, southern Oromia, central parts of Ethiopia, a few parts of the SNNPR region, Dire Dawa, and Harar. Analysis of Fig. 8A also indicates a weak negative association between educated women in most parts of Gambella, some parts of the western Oromia region, and Somalia and the utilization of four or more ANC visits. Furthermore, most parts of the Tigray, parts of the northern Amhara regions, and Benishangul-Gumuz were dominated by a significant negative association between poor women and the utilization of four or more ANC visits, as was evidenced by a coefficient ranging from − 0.275 to 0.122 (Fig. 8B).

In addition, women who had media access were positively associated with the utilization of four or more ANC visits, with the coefficient ranging from 0.410 to 1.010. Media access was a strong predictor of the utilization of four or more ANC visit*s* in many districts of the Tigray, parts of northern Amhara, and Benishangul-Gumuz regions. On the other hand, the positive and weaker relationship between media access and utilization of four or more ANC visit*s* was observed in Dire Dawa, most parts of Somali, Bale, Guji, west and east Hararge of Oromia region Gambela, and the western part of SNNPR region (Fig. 8C). Furthermore, having under-five children shows a weaker positive association with the utilization of four or more ANC visits, with the coefficient ranging from 0.223 to 0.434 in southern and western parts of Ethiopia (Fig. 8D). Conversely, women with under-five children were negatively associated with the utilization of four or more ANC visit*s* in the western part of Tigray, the North Wollo of Amhara, Afar, east Hararge of the Oromia region, a few parts of Gambela, and most parts of the Somali region.

#### Factors associated with the spatial variation of birth attended in a health facility

**Fig. 9:**
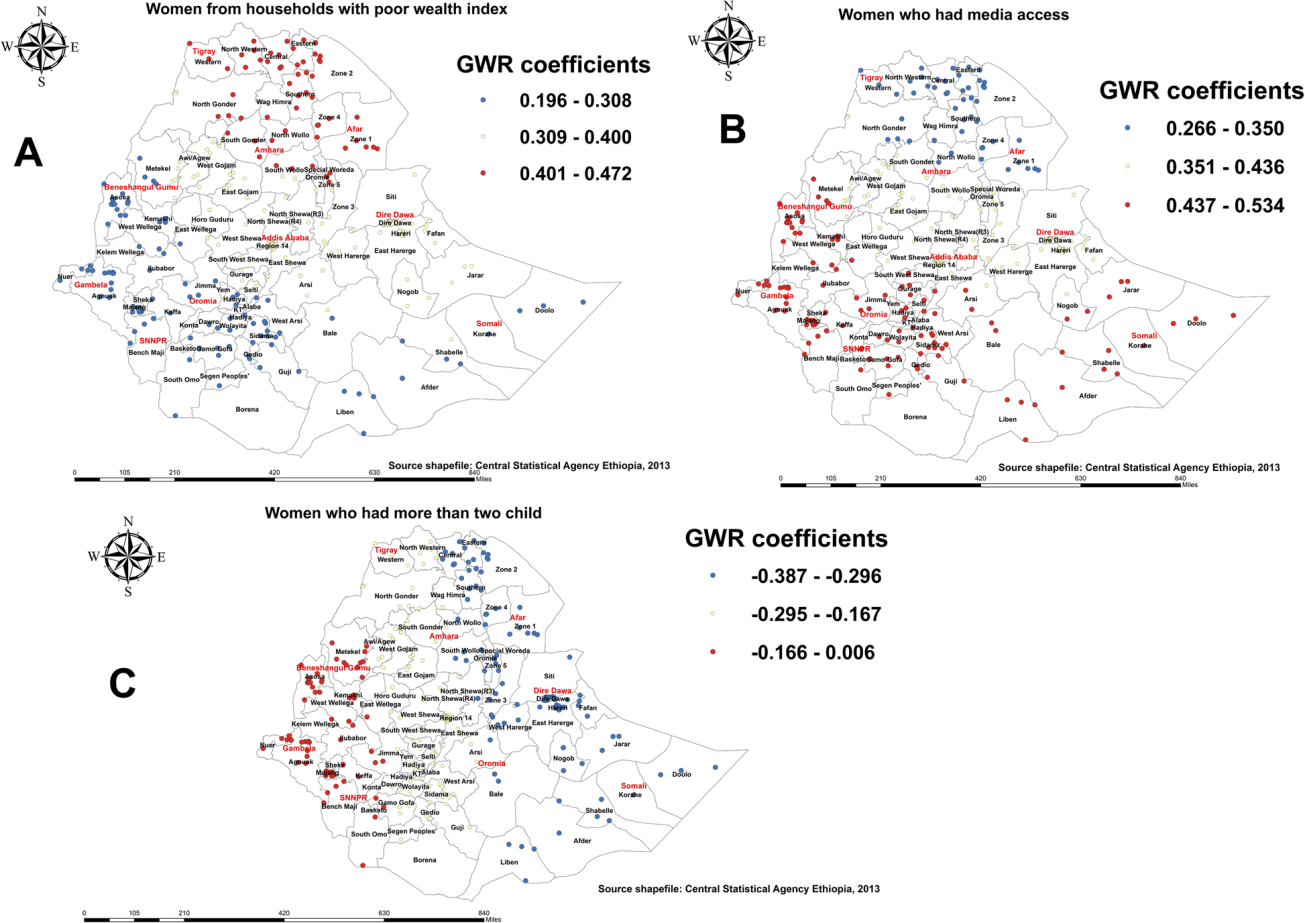
Geographically weighted regression coefficient estimates for predictors of birth attended in a health facility in Ethiopia

GWR analysis revealed the household wealth index, media access, and the number of under-five children in the household were strong and weak predictors of births attended in a health facility among districts in Ethiopia (Table 3; Fig. 9).

As shown in panel A of Fig. 9, women living in poor households were predominantly positively associated with births attended in a health facility, mainly in the Tigray and Afar regions but less so in the Amhara region. Media access was predominantly positively related to pregnant women’s giving birth in a health facility, with the coefficient ranging from 0.266 to 0.534. This was observed in districts of Benishangul-Gumuz, Gambella, the southern and western parts of Oromia, and the Somali region, as shown in Fig. 9B. Furthermore, having more than two children in the household significantly affected the spatial variation in the magnitude of births attended at a health facility, but in a negative direction. Accordingly, the proportion of households with more than two children decreases the number of births attended in a health facility by pregnant women in the Benishangul-Gumuz and Gambella regions (Fig. 9C), with a coefficient ranging from − 0.387 to 0.006.

## Discussion

This study aimed to describe and explain spatial variation in maternal care utilization (pregnant women who received no ANC service from a skilled provider, utilization of four or more ANC visits, and births attended in a health facility) in Ethiopia, using four consecutive national surveys (2005–2019). The Moran’s I statistics established the existence of substantial geographical variation in maternal care utilization across the country. This highlights that geography plays a substantial role in the variation of maternal care utilization among pregnant women in Ethiopia.

The findings showed that maternal care utilization indicators were clustered spatially at the district level. According to Getis-Ord Gi* findings, significant clusters of pregnant women who received no ANC service from a skilled provider were consistently observed in the districts of the Somali region compared to other regions, as well as in some pocket areas of Northern and Southern Ethiopia. These areas are consistent with the predicted maps from the spatial interpolation analysis. Different studies also reported the presence of geographical variations for pregnant women who received no ANC service from a skilled provider [22, 25]. This could be attributed to cultural variation and the nomadic nature of life, especially in the Somali region. Further, the discrepancy could be due to health care coverage differences, and in Ethiopia’s northern regions, a lack of stability could engulf this. Furthermore, this study revealed that the utilization of four or more ANC visits was mainly clustered in Ethiopia’s central parts. Different studies also revealed the presence of geographical variation in the utilization of four or more ANC visits [20, 22, 25]. The most convenient explanation for this is that residents of metropolitan areas have improved infrastructure and better access to healthcare services and media.

A clear spatial pattern of low rates of birth attended in a health facility was observed consistently in parts of the Amhara region (bordering with Afar and Tigray regions), southern parts of Tigray, southern parts of Oromia, some parts of the Gambella region, a few parts of Benishangul-Gumuz and SNNPR regions, and also most districts of the Somali region. For births attended in a health facility, the analysis of hot spots fits with predicted areas identified by kinringing interpolation. Therefore, predicted areas with higher inequalities in maternal care should be considered for potential interventions. These findings are consistent with the evidence of regional variance in institutional delivery in Ethiopia [28, 33] and other settings.

In spatial regression, lack of formal education and having under-five children were positively associated with the spatial clustering of pregnant women who received no ANC service from a skilled provider. Furthermore, women’s education, wealth index, media access, and having under-five children were spatial predictors of utilizing four or more ANC visits in the observed geographic areas. Previous evidence in the literature also indicates that educated women are more likely to utilize ANC care [25, 28], recognizing that those who are educated are in a better position than those who are not educated in terms of improving maternal care utilization. Similarly, women who had access to the media were positively associated with the utilization of four or more ANC visits. Different studies conducted in Ethiopia [22, 26, 28] and other settings [14, 15, 19, 29, 54] also revealed the existence of considerable significant differences in ANC utilization predictors across a geographic area.

Media access and the wealth index exhibited a predominantly positive influence on pregnant women’s decision to give birth in a health facility. Our findings show that pregnant women with a poor wealth index were more likely to give birth in a health facility. In addition, having more than one under-five child in the household was negatively associated with giving birth in a health facility in various districts. Similarly, a study conducted in Sub-Saharan Africa [31], Nigeria [35], and Rwanda [19] identified the number of children as a significant predictor of local geographical variation in health facility delivery.

Overall, the maternal care utilization analysis of hot spot areas using Getis-Ord Gi predicted areas from kringing interpolation fits with the analysis of spatial regression somewhere and varies also somewhere. This study makes several contributions to the literature about inequalities in maternal care utilization among pregnant women, particularly in terms of spatial predictors. Furthermore, this research has several strengths. To begin with, the study was based on large, nationally representative datasets and hence has sufficient statistical power. Further, the study’s estimates were done after the data were weighted for probability sampling and nonresponse to make them representative at national and regional levels. Therefore, it can be generalized to all pregnant women in the study setting. Also, the spatial regression modeling approaches were applied to understand the pattern and geographical predictors of the observed variation in maternal care utilization.

However, the current study has various limitations that must be noted when evaluating the results. The first issue to consider is that the cross-sectional nature of the study’s design makes it impossible to infer causation between the outcomes and the predictors considered in this study. Second, clusters with missing longitude and latitude data in the dataset were excluded from the spatial analysis, which could affect the generalizability of the findings. Further, due to the DHS sampling strategy, there may be a bias in the detection hotspot. Despite these limitations, we believe that our findings and suggestions will contribute immensely to a better understanding of inequalities in maternal care utilization in Ethiopia.

## Conclusion

Using four consecutive national surveys (2005–2019), this paper described and explained spatial variation in maternal care utilization in Ethiopia. Pregnant women’s utilization of maternal care services varies geographically across regions. It is, therefore, vital to plan to combat maternal care inequalities in a manner suitable for district-specific variations.

These findings suggest intervention strategies should address the context of geographic areas where a high proportion of women did not receive any ANC service from a skilled provider, areas with low hotspots of the utilization of four or more ANC visits, and health facility delivery. Furthermore, predictors of geographical variation identified during spatial regression analysis can inform efforts to achieve geographical equity in maternal care utilization. Further, there is also a need to identify as-yet-unidentified predictors that might account for the unexplained geographical inequality of maternal care.

## Supplementary Information

**Additional file 1.** Spatial Autocorrelation report.

## Data Availability

All the data that were used in this study are free and publicly available in the MEASURE DHS program, anyone can access and utilize the data from www.measuredhs.com.

## Abbreviations

ANC: Antenatal care
CSA: Central Statistics Agency
DHS: Demographic and Health Survey
EDHS: Ethiopia Demographic and Health Survey
MMR: Maternal Mortality Rate
SNNPR: Southern Nations, Nationalities, and Peoples' Region
GWR: Geographically Weighted Regression
OLS: Ordinary Least Square method
