# Supplementary material for: Identifying geographical inequalities of maternal care utilization in Ethiopia: a Spatio-temporal analysis from 2005 to 2019
Source: BMC Health Serv Res. 2022 Nov 30;22:1455. doi: 10.1186/s12913-022-08850-1 (PMC9714149; doi:10.1186/s12913-022-08850-1)
Supplement: Supplementary file 1 — Additional file 1. Spatial Autocorrelation report. [file 12913_2022_8850_MOESM1_ESM.docx]

| 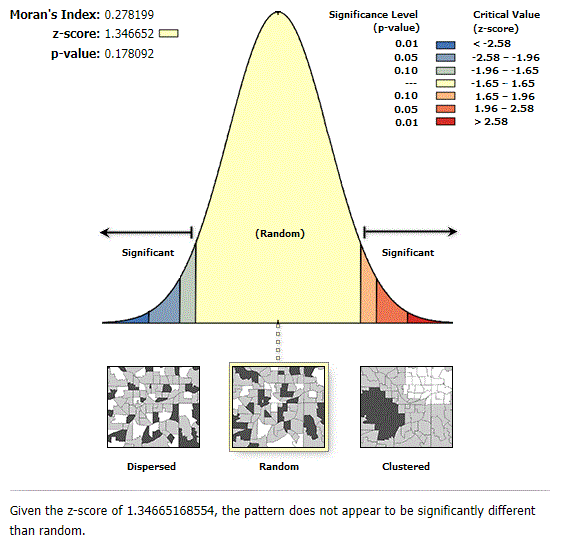  **A** | 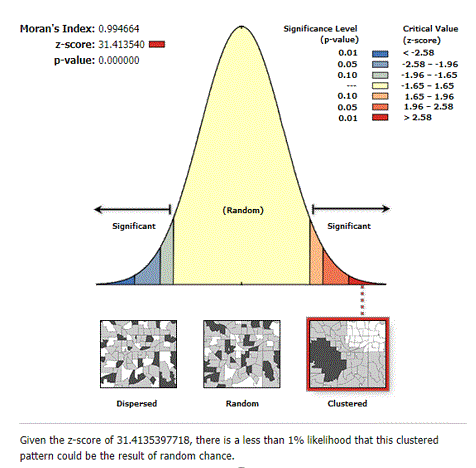  **B** |
| --- | --- |
| 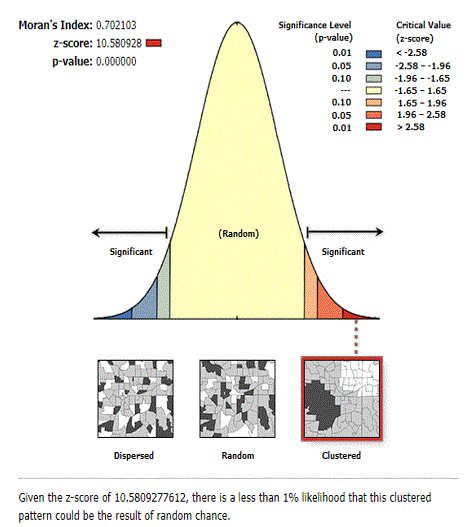  **C** | 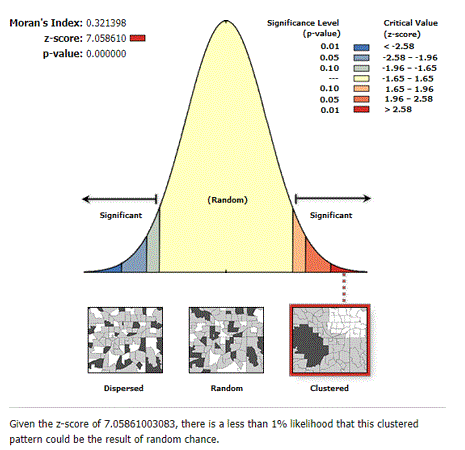  **D** |

**Supplementary figure 1:** The global spatial autocorrelation of pregnant who received no antenatal care(ANC) service from a skilled provider in Ethiopia: 2005 (A), 2011 (B), 2016 (C), and 2019 (D)

| 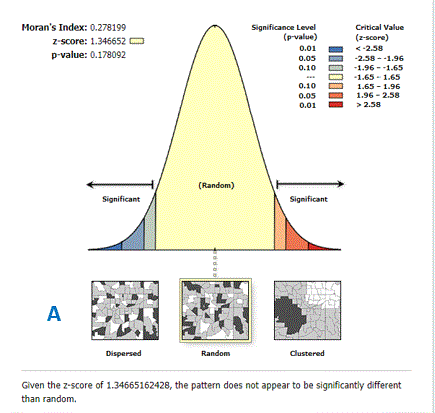  **A** | 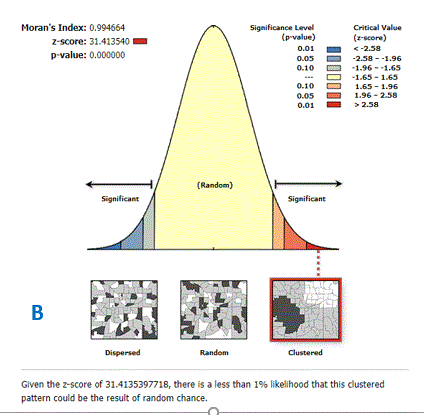  **B** |
| --- | --- |
| 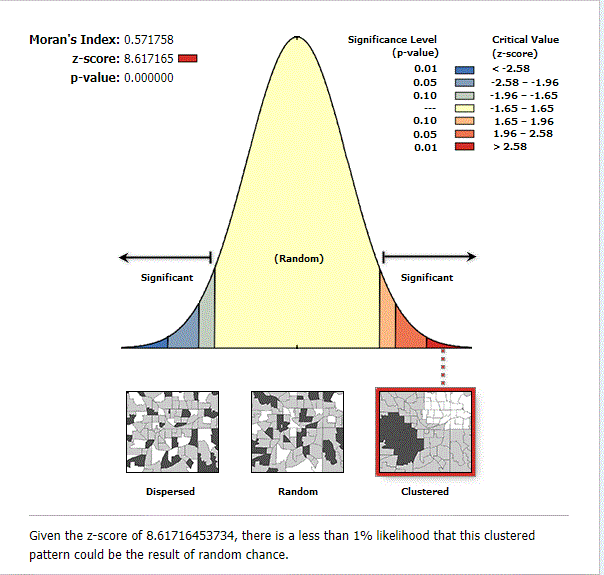  **C** | 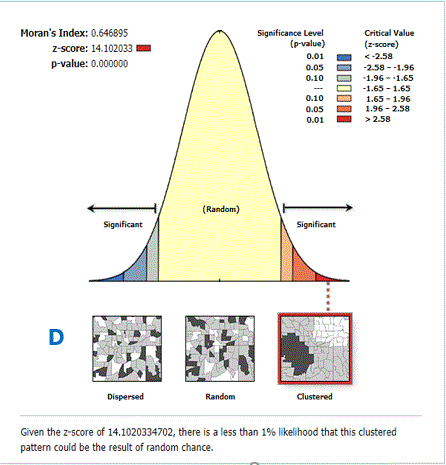  **D** |

**Supplementary figure 2:** The global spatial autocorrelation of utilization of four or more antenatal care (ANC) visit among pregnant women in Ethiopia: 2005 (A), 2011 (B), 2016 (C), and 2019 (D)

**Supplementary figure 3:** The global spatial autocorrelation of birth attended in a health facility in Ethiopia: 2005 (A), 2011 (B), 2016 (C), and 2019 (D)

| 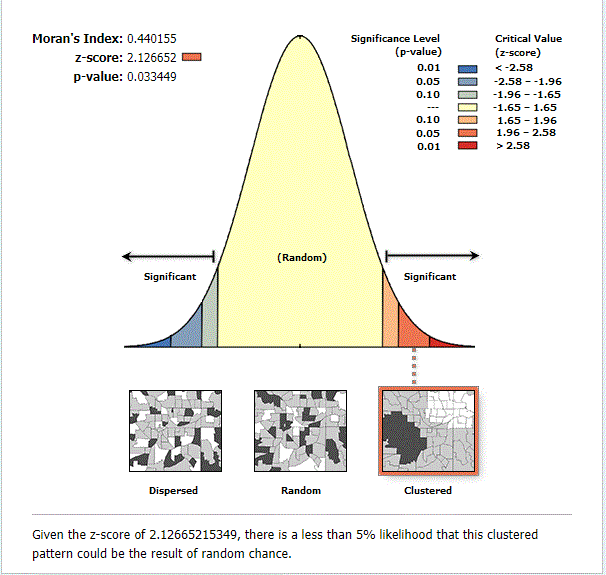  **A**  **A)** | 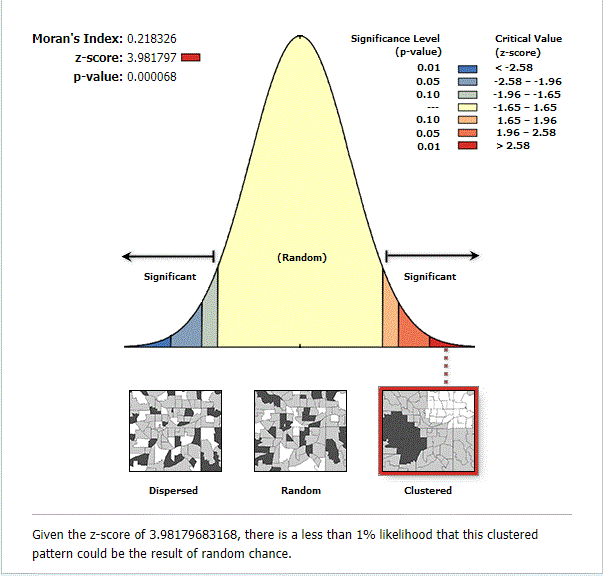  **B** |
| --- | --- |
| 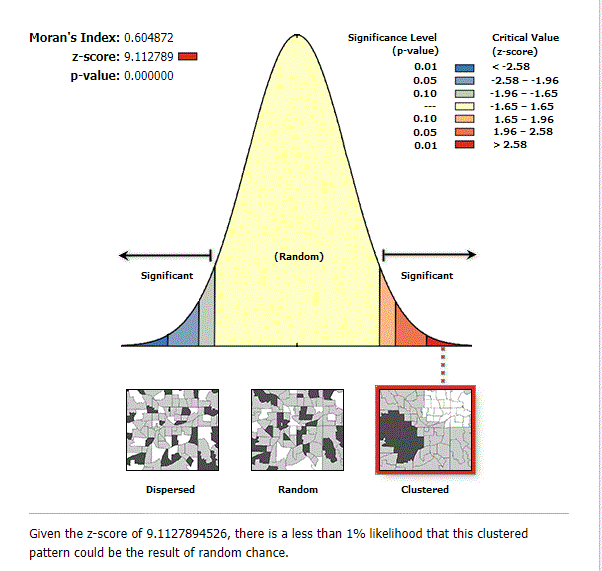  **C** | 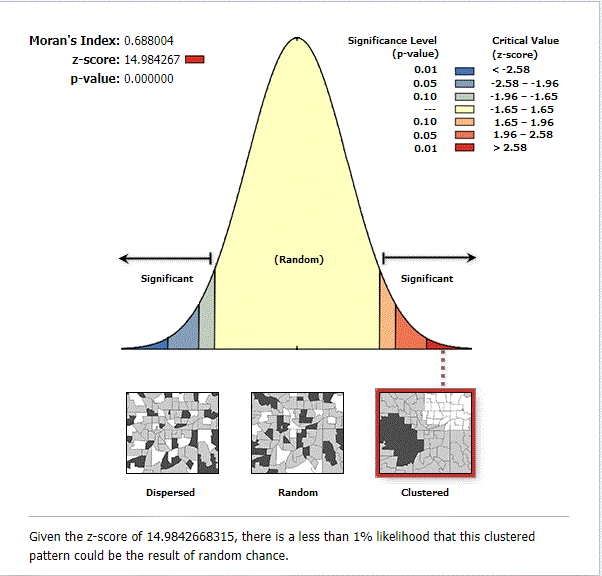  **D** |
